# *Acinetobacter baumannii* utilizes a novel protective factor to combat desiccation-induced oxidative stress

**DOI:** 10.1371/journal.pone.0350814

**Published:** 2026-06-03

**Authors:** John M. Farrow, Greg Wells, Everett C. Pesci

**Affiliations:** Department of Microbiology and Immunology, The Brody School of Medicine at East Carolina University, Greenville, North Carolina, United States of America; University of Florida, UNITED STATES OF AMERICA

## Abstract

One of the factors that facilitates the nosocomial spread of the frequently multidrug-resistant pathogen *Acinetobacter baumannii* is its ability to persist on dry surfaces for weeks. These bacteria do not form spores, but instead rely on a group of protective factors to survive desiccation. We investigated a protective factor of unknown function, referred to here as desiccation tolerance protein C (DtpC), which we found to be co-transcribed with another protective factor, the KatE catalase. A Δ*dtpC*Δ*katE* mutant strain had a greatly decreased ability to survive desiccation, but complementation with either *dtpC* or *katE* individually could restore survival to the wild-type level, showing that DtpC and KatE are each sufficient for desiccation tolerance. Since KatE also protects cells from oxidative stress, we examined whether oxygen had an effect on desiccation survival, and analyzed the formation of reactive oxygen species in dried cells. Compared to the wild-type strain, the Δ*dtpC*Δ*katE* mutant strain was much more sensitive to the detrimental effects of oxygen during desiccation, and we observed that hydrogen peroxide accumulated in dried cells of the Δ*dtpC*Δ*katE* mutant strain over time, whereas hydrogen peroxide levels were limited when either *dtpC* or *katE* was present. Additionally, either *dtpC* or *katE* was sufficient to limit desiccation-induced mutagenesis, which is driven by DNA damage during drying. By using structural predictions and site-directed mutagenesis, we found that DtpC’s protective activity depends on a domain that is similar to heme-oxygenase-like diiron oxidases. No other proteins in this family have been linked to desiccation tolerance or oxidative stress, making DtpC a novel type of protective factor. Overall, our data show that both DtpC and KatE act to limit desiccation-induced oxidative stress, which is critical for *A. baumannii* to survive on dry surfaces.

## Introduction

*Acinetobacter baumannii* is a challenging gram-negative bacterial pathogen that primarily infects severely ill or injured individuals in healthcare settings [1,2]. It is considered to be a worldwide threat because of extremely high rates of multidrug resistance (MDR). A global study of clinical isolates found that *A. baumannii* had an overall MDR rate of 44%, which was the highest rate among gram-negative bacterial pathogens [3], and in 2018 the World Health Organization designated *A. baumannii* as a top priority for the development of new treatments [4]. This organism also presents a challenge because infections can easily spread to other susceptible patients, especially in intensive care units. Outbreaks typically remain small, but can affect larger numbers of people if appropriate control measures are not taken, or if *A. baumannii* becomes endemic to the hospital environment [5,6]. Difficult conditions in military field hospitals during the Iraq war led to an *A. baumannii* outbreak from 2002 to 2004 that affected over one hundred wounded soldiers [7,8]*.* More recently, in 2020 the United States Centers for Disease Control and Prevention reported a 78% increase in hospital-onset *A. baumannii* infections associated with the large increase in hospitalizations due to COVID-19 [9]. The increased number of infections by *A. baumannii,* and other MDR organisms, during this time period was partially attributed to the breakdown of normal infection control procedures as hospitals dealt with large numbers of COVID-19 patients. These examples highlight the ability of *A. baumannii* to rapidly emerge and spread in hospitals when conditions are favorable.

Some of the properties that make *A. baumannii* difficult to control in healthcare settings include its ability to withstand challenging environmental conditions like starvation and desiccation. This allows *A. baumannii* to persist in the hospital environment and spread via direct contact or through contact with contaminated objects. In particular, *A. baumannii* has a high level of tolerance to desiccation, which is similar to the gram-positive nosocomial pathogen *Staphylococcus aureus* [10]. Clinical *A. baumannii* isolates can commonly survive on dry surfaces for about one month, although some isolates can survive drying for three months or longer [11–13]. During outbreaks, *A. baumannii* is often isolated from dry objects that came in direct contact with infected patients, like bed frames, bedding, and reusable medical equipment, but it has also been found on a wide range of other objects in the hospital environment, including monitors, curtains, fans, furniture, television sets, and computer keyboards [7,11,14–17]. Additionally, it has been isolated from air conditioners and ventilation systems, which are a potential source of airborne spread [7,18]. In one case, the source of an outbreak was traced to contaminated dust inside medical equipment [19]. These observations imply that desiccation tolerance is an important property that supports the persistence and spread of *A. baumannii* in hospitals.

Desiccation is a common stress faced by bacteria in both natural and man-made environments, and it presents multiple challenges to cells. Drying leads to a loss of turgor pressure, and can cause membrane phase change and destabilization [20]. Proteins can also be destabilized under dry conditions and become denatured. DNA damage is frequently seen with desiccation, which is thought to be the result of oxidative stress that occurs during drying, and oxidative damage has also been shown to occur to proteins from dried cells [21–23]. Bacteria have developed different strategies to deal with this combination of different stresses during desiccation. Some bacterial species form spores that are highly resistant to multiple physical and chemical stresses [24]. Non-spore forming bacteria, such as *A. baumannii*, vary widely in their ability to tolerate desiccation, and there does not seem to be any specific response to allow them to deal with this stress. Instead, different species utilize a variety of strategies to survive drying. Exopolysaccharide production is a common defense that helps prevent water loss and protects the cell surface [21,22]. The production of small molecule compatible solutes, especially the disaccharide trehalose, is another common defense. These molecules help protect cells from changes in osmolarity during drying and stabilize cellular constituents [21]. Some species also produce certain types of intrinsically disordered proteins, such as hydrophilins or late embryogenesis abundant type proteins, which prevent protein aggregation within dried cells and stabilize cell components [22]. Oxidative stress defenses are induced in multiple bacterial species during drying [25–29]. Yet while many bacteria have the capability to express some of these defenses, it is unclear why certain species, or sometimes even different strains of the same species, have very different capacities to tolerate desiccation.

**Fig 1:**
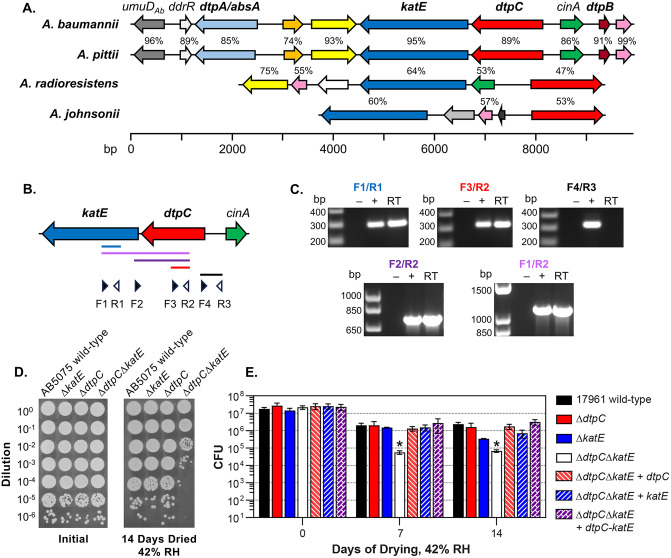
The *dtpC* and *katE* genes form an operon in *A. baumannii* that is important for desiccation tolerance. **(A)** A diagram showing the relative locations of the *dtpC* and *katE* genes in four representative *Acinetobacter* species. *A. pittii* is a member of the *A. baumannii-calcoaceticus* complex. Orthologous genes are shown in the same color. The amino acid identity of each orthologous protein to the *A. baumannii* protein is shown above the respective gene. **(B)** A diagram of the *dtpC* and *katE* genes, showing the relative positions of primers used for RT-PCR analysis, and the positions of expected PCR products. **(C)** PCR reactions using either RNA alone as a control (–), chromosomal DNA (+), or reverse-transcribed RNA (RT) as a template were analyzed by agarose gel electrophoresis. The primer set used is indicated above each photograph. These data are representative of two independent experiments. **(D)** and **(E)** The survival of strains before and after desiccation at 42% relative humidity was assessed. The strains tested were either the wild-type strain AB5075 in (D) or ATCC 17961 in (E); Δ*dtpC*, Δ*katE*, and Δ*dtpC*Δ*katE* deletion mutants in each respective wild-type background; and the Δ*dtpC*Δ*katE* deletion mutant complemented by insertion of either *dtpC, katE,* or *dtpC-katE* onto the chromosome. The data in (D) are representative of three independent experiments. The data presented in (E) are the mean ± SD from at least three independent experiments (for Δ*katE*, *n* = 3; for 17961 wild-type, Δ*dtpC,* and Δ*dtpC*Δ*katE, n* = 4; for all other strains *n* = 5). For each timepoint, the CFU of each strain was compared to wild-type by the Kruskal-Wallis test with Dunn’s multiple comparisons post-test. *p < 0.05.

The high level of desiccation tolerance exhibited by *A. baumannii* appears to involve the combined effects of multiple factors. Factors that protect the surface of the cell include the regular incorporation of hepta-acylated lipid A into the outer membrane, which typically occurs as a stress response in other species, and helps to reinforce the membrane from drying [30]. *A. baumannii* can also produce a polysaccharide capsule that provides some protection from desiccation [31]. As for factors that protect other cellular constituents, RecA-dependent DNA damage repair responses appear to be important to mitigate desiccation-induced DNA damage [32,33]. Other protective factors appear to be induced by general stress responses, as stationary phase *A. baumannii* cells have a greatly improved capacity to tolerate desiccation compared to actively growing cells [13,34,35]. We found that the two-component response regulator BfmR was essential for controlling these responses in *A. baumannii* strain ATCC 17961 [13]. Interestingly, one cluster of genes that had greatly decreased expression in a Δ*bfmR* mutant strain encodes multiple factors that support desiccation tolerance [36]. This gene cluster is conserved in other closely related species in the *A. baumannii-calcoaceticus* complex, which includes the *Acinetobacter* species that are responsible for most human infections [35]. Two genes in this cluster encode hydrophilins, *dtpA* (also referred to as *absA*) and *dtpB*, which support desiccation tolerance and potentially stabilize cellular enzymes during drying [35]. The KatE catalase is also encoded in this cluster, which we found could support long-term drying survival [13]. Additionally, an *A. baumannii* strain with a transposon inserted into the gene immediately upstream from *katE* had greatly reduced desiccation tolerance. Expression of the protein encoded by this gene (ABUW_2437 in *A. baumannii* strain AB5075), which we will refer to as desiccation tolerance protein C (DtpC), in the less desiccation-tolerant strain ATCC 17978 improved its ability to survive drying, showing that DtpC had some protective activity [34]. We also noted that the *dtpC* and *katE* genes are found in close proximity in the genomes of other *Acinetobacter* species that are not part of the *A. baumannii-calcoaceticus* complex, including the highly desiccation-resistant species *A. radioresistens* (Fig 1A). In this study, we investigated the protective function of DtpC and its relationship with KatE. We found that DtpC and KatE act in parallel to combat oxidative stress in dried cells. Our data show that preventing desiccation-induced oxidative stress is critical for *A. baumannii* desiccation tolerance, and that DtpC is a novel type of protective factor that supports the persistence of *A. baumannii* on dry surfaces.

## Results

### The *dtpC* and *katE* genes form an operon that is important for *A. baumannii* desiccation tolerance

Data from previous studies showed that both the *dtpC* and *katE* genes could aid the survival of *A. baumannii* cells during desiccation [13,34]. However, some of these data were generated using a strain with a transposon inserted into *dtpC*, and it was unclear if this insertion also affected *katE* expression because the orientation of *dtpC* and *katE* on the *A. baumannii* chromosome suggested that these genes could be co-transcribed as an operon. In *A. baumannii,* the *dtpC* gene is encoded upstream from *katE*, and the coding regions of the two genes are separated by 54 bp (Fig 1B). Additionally, a global transcriptome mapping study in *A. baumannii* strain ATCC 17978 identified two potential transcriptional start sites upstream from *dtpC*, but did not identify any start sites upstream from *katE*, suggesting that there is a single promoter for these two genes [37]. To determine if *dtpC* and *katE* are encoded on the same mRNA, we extracted RNA from *A. baumannii* strain ATCC 17961 and performed reverse transcription followed by PCR with oligonucleotide primers that would amplify DNA fragments either within *dtpC* or *katE*, or DNA fragments that spanned both genes. We observed that fragments that extended from *dtpC* into the *katE* coding region could be amplified from reverse-transcribed RNA, whereas a fragment that extended from the 5’-end of *dtpC* into the non-coding upstream region could not be amplified (Fig 1C). These results confirmed that *dtpC* and *katE* are co-transcribed as a two gene operon.

To assess the individual and combined importance of *dtpC* and *katE* for *A. baumannii* desiccation tolerance, we constructed in-frame deletion mutations in either *dtpC* or *katE*, and a Δ*dtpC*Δ*katE* double mutation. These mutations were introduced into two highly desiccation-tolerant *A. baumannii* strains, AB5075 and ATCC 17961. For experiments to examine the effects of these mutations on desiccation tolerance, cells from overnight cultures of each strain were washed and suspended in deionized water, and then 5 µl samples of cell suspensions were air-dried on a polystyrene surface (approximately 1 h at room temperature) before being stored in the dark at 25°C and a relative humidity (RH) of 42%. This approach was meant to mimic some aspects of bacterial contamination of plastic surfaces in the hospital environment, while removing exogenous proteins or salts that could impact the results [10]. The deletion of *dtpC* alone did not affect drying survival, while the deletion of *katE* alone appeared to cause a small decrease in survival after 14 days of desiccation (Fig 1D and 1E), which was similar to our previous observations [13]. In contrast, there was a large decrease in the survival of the Δ*dtpC*Δ*katE* mutant strain after one week of desiccation. For strain ATCC 17961, the Δ*dtpC*Δ*katE* mutant strain had approximately a two log decrease in the number of colony forming units (CFU) recovered after seven days of drying, which corresponded to an approximately 45-fold lower survival rate than the wild-type strain (Fig 1E). The Δ*dtpC*Δ*katE* mutant in strain ATCC 17961 was also complemented by introducing an intact copy of either *dtpC* alone, *katE* alone, or both *dtpC* and *katE*, into the *att*Tn7 site of the *A. baumannii* chromosome. Each of the complementing DNA fragments included the native promoter sequences upstream from *dtpC* to drive gene expression. For complementation with *katE* alone, a premature stop codon was introduced at codon 14 of the *dtpC* coding sequence so that *katE* could be expressed from its native promoter. When either *dtpC* or *katE* was supplied to the Δ*dtpC*Δ*katE* mutant strain, the drying survival was restored to a level similar to the wild-type strain (Fig 1E). Complementation of the Δ*dtpC*Δ*katE* mutant with both *dtpC* and *katE* together had a similar effect to supplying each gene individually (Fig 1E). To further confirm that *katE* alone could restore desiccation tolerance, a copy of *katE* under the control of the *trc* promoter was introduced into the Δ*dtpC*Δ*katE* mutant strain on a plasmid. The plasmid-borne copy of *katE* was also able to restore the drying survival of the Δ*dtpC*Δ*katE* mutant strain to the wild-type level (S1 Fig). These results showed that the *dtpC* and *katE* genes are each sufficient for desiccation tolerance in *A. baumannii*. However, if both genes are removed then desiccation tolerance is greatly reduced.

### DtpC is predicted to be a heme-oxygenase-like diiron oxidase

**Fig 2:**
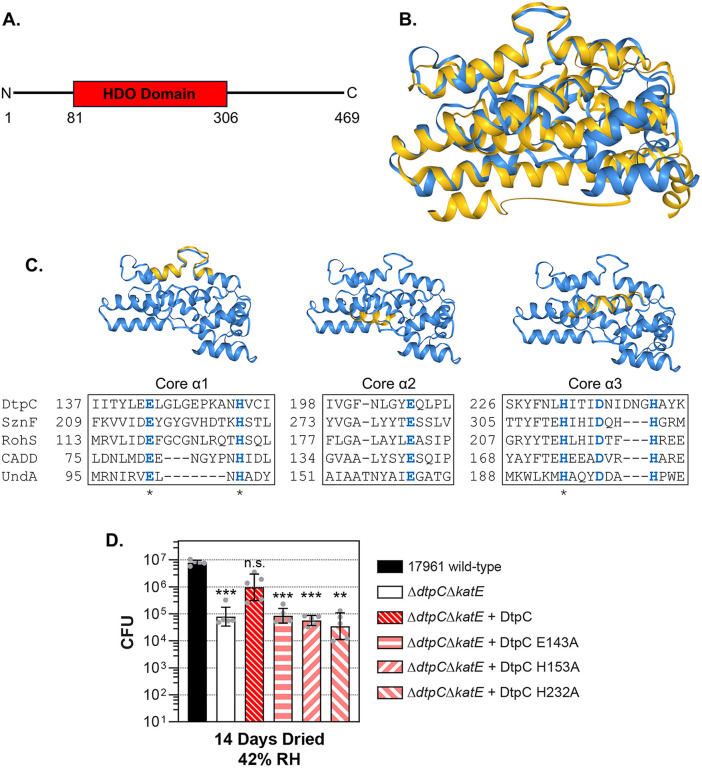
Mutations in a predicted heme-oxygenase-like diiron oxidase (HDO) domain disrupt the protective activity of DtpC. The structure of DtpC predicted by AlphaFold was used for a structural similarity search on the Foldseek search server. **(A)** A graphic showing the portion of DtpC with similarity to HDOs. **(B)** An overlay of the potential HDO domain from the predicted structure of DtpC (yellow) (AlphaFold D0C8B3) with the crystal structure of SznF (blue) (PDB 6VZY) that show significant similarity; TM-Score: 0.57818, RMSD: 8.43. Images were generated by Foldseek. **(C)** A structure-based sequence alignment of the core HDO domain α–helices from DtpC with other HDO family proteins that have known structures. The relative position of each α-helix is highlighted in the ribbon structure above each alignment. Key amino acid residues for binding metal co-factors are shown in blue type. Amino acid residues that were mutated are marked with an asterisk. **(D)** Survival of either the wild-type strain ATCC 17961, the Δ*dtpC*Δ*katE* deletion mutant, or the Δ*dtpC*Δ*katE* deletion mutant complemented with either the wild-type *dtpC* allele, or mutated *dtpC* alleles that introduced amino acid substitutions into DtpC, were assessed by CFU counts after drying and incubation at 25°C and 42% RH for 14 days. The data presented are the mean ± SD from five independent experiments. The mean CFU for each strain was compared to the wild-type by Welch’s ANOVA with Dunnett’s multiple comparisons post-test on log_10_-transformed data. **p < 0.01, ***p < 0.001, n.s. = not significant.

To learn more about the protective activity of the protein encoded by *dtpC*, we examined the similarity of DtpC to other proteins. BLAST searches showed that proteins with the highest similarity to the primary amino acid sequence of *A. baumannii* DtpC were found in other *Acinetobacter* species, and some *Stutzerimonas* and *Pseudomonas* species, but this did not provide much insight since none of these proteins have been characterized. Another approach to find proteins with similar or related functions is to compare their three-dimensional structure. Since the structure of DtpC has not been solved experimentally, we used the predicted structure of *A. baumannii* DtpC that is available in the AlphaFold Protein Structure Database [38,39]. We compared the predicted structure of DtpC to other proteins using the FoldSeek Search Server [40], and focused on structurally similar proteins with experimentally verified structures. These results predicted that DtpC contains a domain that is structurally similar to several heme-oxygenase-like diiron oxidases (HDOs) (Fig 2A and 2B). The function of the majority of the proteins in this family are not known, but the HDOs that have been studied are enzymes involved in the biosynthesis of small molecules. These proteins contain an α-helical bundle that coordinates two metal ions, which are typically iron, that are used to activate molecular oxygen as part of their catalytic mechanism. We aligned the amino acid sequences of DtpC based on their structural positions to the corresponding amino acids in four HDO family proteins: the N-oxygenases SznF [41], and RohS [42]; the *Chlamydia* protein associating with death domains (CADD), which synthesizes *para*-aminobenzoate [43]; and the fatty acid decarboxylase UndA [44]. This alignment showed that six key amino acids involved in coordinating the metal cofactors in other HDOs [43–46] are predicted to be present in the same three-dimensional positions in DtpC (Fig 2C). For SznF and UndA, alanine substitutions for three of these amino acids, corresponding to E143, H153, and H232 in DtpC, each individually disrupted the activity of these enzymes, and mutating all three in combination disrupted the activity of CADD [44,45,47]. To determine if these amino acids were important for the protective function of DtpC, we made mutations in the *dtpC* gene that would convert either glutamate 143, histidine 153, or histidine 232 to alanine. We then transferred these mutated *dtpC* alleles, with the native *dtpC* promoter, onto the chromosome of the Δ*dtpC*Δ*katE* mutant strain and tested to see if the mutated forms of DtpC could restore desiccation tolerance. After 14 days of drying at 42% RH the survival of the Δ*dtpC*Δ*katE* mutant strain was significantly reduced compared to the wild-type strain, and complementation of the Δ*dtpC*Δ*katE* mutant with the wild-type *dtpC* improved survival to a level that was not significantly different from the wild-type (Fig 2D). In contrast, complementation of the Δ*dtpC*Δ*katE* mutant strain with each of the mutated *dtpC* alleles did not improve the survival of the Δ*dtpC*Δ*katE* mutant (Fig 2D). These results showed that the E143A, H153A, and H232A substitutions each disrupted the protective activity of DtpC, suggesting that the HDO domain of DtpC is important for its protective activity in desiccated cells.

### The Δ*dtpC*Δ*katE* mutant strain is more susceptible to oxygen-mediated killing during desiccation

Next, we began to consider how DtpC and KatE might function to protect cells during desiccation. The function of KatE is to defend cells from oxidative stress by scavenging hydrogen peroxide, and this enzyme is the major source of catalase activity in stationary phase *A. baumannii* cells [48]. The fact that KatE alone could protect dried cells suggested that limiting oxidative stress is a key facet of *A. baumannii* desiccation tolerance. Since DtpC is capable of protecting dried cells when KatE was absent, we hypothesized that DtpC also acts to defend cells from desiccation-induced oxidative stress. If this was the case, then *dtpC* and *katE* should be unnecessary under conditions where the production of reactive oxygen species (ROS) is limited, such as when molecular oxygen is not present. To test this, samples of cells from the wild-type, Δ*dtpC*, Δ*katE*, and Δ*dtpC*Δ*katE* mutant strains were allowed to air-dry under normal conditions, and then the dried cells were incubated in an anaerobic chamber in order to limit ROS formation during desiccation. The humidity in the anaerobic chamber was extremely low (< 5% RH), so to account for this difference we incubated paired sets of dried cell samples under the same conditions of temperature and humidity as in the anaerobic chamber, but with normal atmospheric oxygen levels. After 14 days, the dried samples were removed from the anaerobic chamber for rehydration and plating to determine survival.

**Fig 3:**
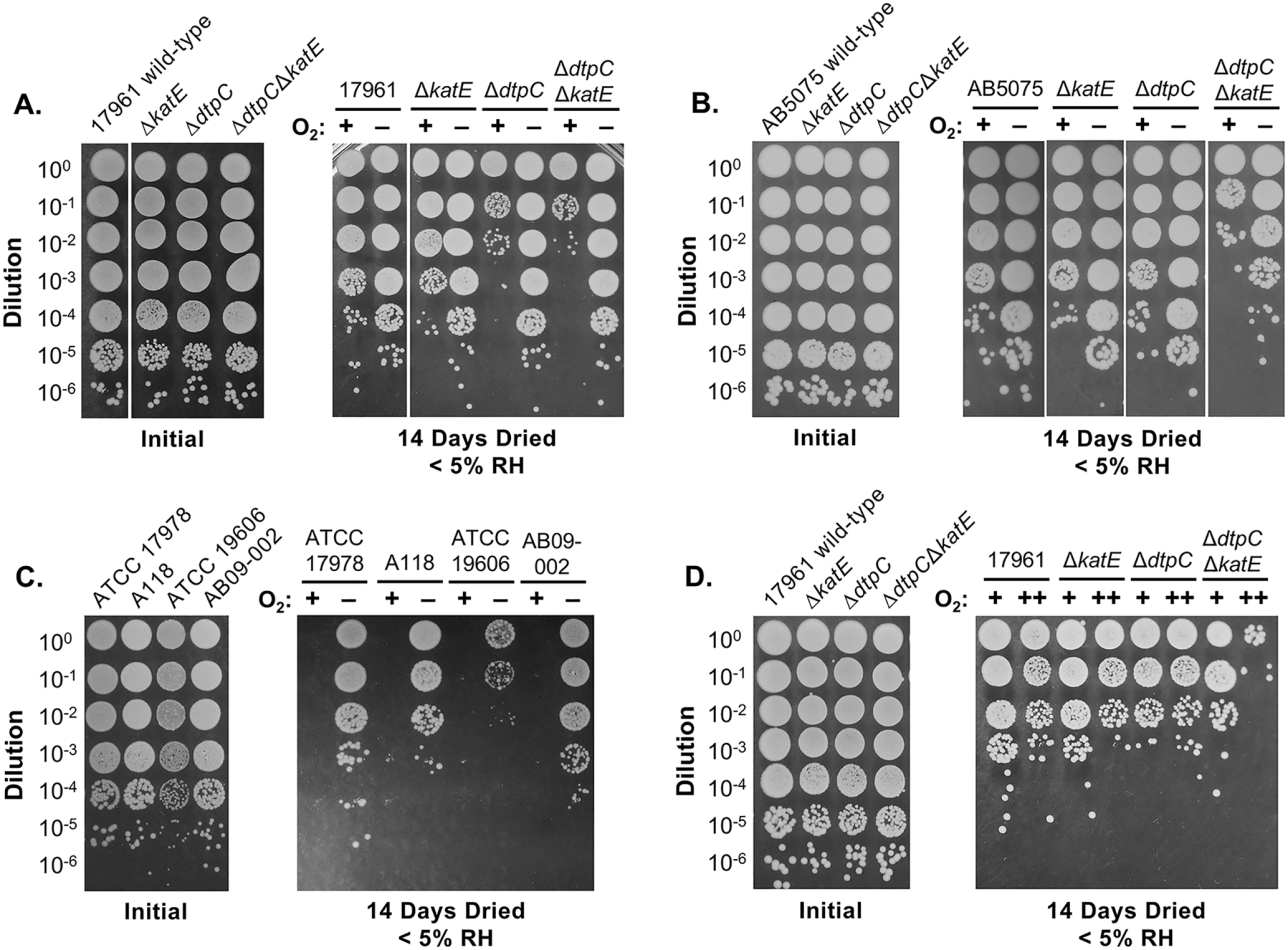
Limiting oxygen levels during desiccation improves the survival of the Δ*dtpC*Δ*katE* mutant strain. Samples of either *A. baumannii* wild-type strains ATCC 17961 **(A)** or AB5075 **(B)**; Δ*dtpC*, Δ*katE*, and Δ*dtpC*Δ*katE* deletion mutants in the respective wild-type backgrounds; or other less drying-resistant *A. baumannii* wild-type strains **(C)** (Table 1), were diluted and plated either prior to drying, or after drying and incubation at 25°C and < 5% RH for 14 days. In panels **(A)**, **(B)**, and **(C)**, dried cells were incubated with either atmospheric O_2_ levels (+) or in an anaerobic chamber (–). In panel **(D)**, dried cells were incubated with either atmospheric O_2_ levels (+) or in a container that was flushed with O_2_, then sealed (++). These data are representative of three independent experiments. Images in panels (A) and (B) were cropped or edited for consistency in formatting. The original versions of these images are supplied in S2 Fig.

The first thing we observed was that the survival of all strains, including the wild-type strain ATCC 17961, improved when dried cells were incubated in the absence of oxygen versus in the presence of oxygen (Fig 3A). Most interestingly, in the absence of oxygen the survival of each of the mutant strains, including the Δ*dtpC*Δ*katE* mutant strain, was similar to the wild-type strain (Fig 3A). For the Δ*dtpC*Δ*katE* mutant strain, this appeared to cause an approximately 1000-fold increase in survival without oxygen compared to survival in the presence of oxygen (Fig 3A). We also performed these experiments with the Δ*dtpC*, Δ*katE*, and Δ*dtpC*Δ*katE* mutants in strain AB5075 and observed similar results (Fig 3B). With respect to oxygen availability, the only difference we noticed between strains ATCC 17961 and AB5075 was that for strain AB5075, we observed that the Δ*dtpC*Δ*katE* mutant strain had reduced survival even when oxygen was absent (Fig 3B). Even so, the survival of the AB5075 Δ*dtpC*Δ*katE* mutant strain appeared to be greatly improved when oxygen was removed, with what appeared to be an approximately 100-fold increase in survival without oxygen versus in the presence of oxygen (Fig 3B). These results showed that the decreased viability of the Δ*dtpC*Δ*katE* mutant strain during desiccation was due to oxygen-dependent effects.

**Table 1:** *A. baumannii* strains and plasmids used in this study.

Strain or Plasmid	Description	Reference or source
		
*A. baumannii* strains		
ATCC 17961	Clinical isolate from blood	ATCC
17961-Δ*katE*	*katE* deletion mutant derived from strain ATCC 17961	[13]
AbGW-Δ*dtpC*	*dtpC* deletion mutant derived from strain ATCC 17961	This study
AbGW-Δ*dtpC*Δ*katE*	*dtpC katE* double deletion mutant derived from strain ATCC 17961	This study
AbGW-Δ*dtpC*Δ*katE-dtpC*	AbGW-Δ*dtpC*Δ*katE* with *dtpC* inserted at the *att*Tn7 site	This study
AbGW-Δ*dtpC*Δ*katE-dtpC-*Stop-*katE*	AbGW-Δ*dtpC*Δ*katE* with *dtpC* 40C > T and *katE* inserted at the *att*Tn7 site	This study
AbGW-Δ*dtpC*Δ*katE-dtpC*-*katE*	AbGW-Δ*dtpC*Δ*katE* with *dtpC-katE* inserted at the *att*Tn7 site	This study
AbGW-Δ*dtpC*Δ*katE-dtpC-*E143A	AbGW-Δ*dtpC*Δ*katE* with *dtpC* 428–429AG > CA inserted at the *att*Tn7 site	This study
AbGW-Δ*dtpC*Δ*katE-dtpC-*H153A	AbGW-Δ*dtpC*Δ*katE* with *dtpC* 457–458CA > GC inserted at the *att*Tn7 site	This study
AbGW-Δ*dtpC*Δ*katE-dtpC-*H232A	AbGW-Δ*dtpC*Δ*katE* with *dtpC* 694–695CA > GC inserted at the *att*Tn7 site	This study
AB5075	Military isolate from osteomyelitis	[66]
AB5075-Δ*dtpC*	*dtpC* deletion mutant derived from strain AB5075	This study
AB5075-Δ*katE*	*katE* deletion mutant derived from strain AB5075	This study
AB5075-Δ*dtpC*Δ*katE*	*dtpC katE* double deletion mutant derived from strain AB5075	This study
ATCC 17978	Clinical isolate from spinal meningitis	ATCC
ATCC 19606	Clinical isolate from urine	ATCC
A118	Clinical isolate from blood	[67]
AB09−002	Military isolate from wound culture	[13]
		
Plasmids		
pEX18Ap	Suicide vector, Amp^R^	[68]
pEX18Tc	Suicide vector, Tc^R^	[68]
pΔ*dtpC*-suc	Suicide plasmid carrying *dtpC* deletion, Amp^R^	This study
pΔ*dtpC*Δ*katE*-suc	Suicide plasmid for deletion of *dtpC-katE*, Amp^R^	This study
pTcΔ*dtpC*-suc	Suicide plasmid carrying *dtpC* deletion, Tc^R^	This study
pTcΔ*katE*-suc	Suicide plasmid carrying *katE* deletion, Tc^R^	This study
pTcΔ*dtpC*Δ*katE*-suc	Suicide plasmid for deletion of *dtpC-katE*, Tc^R^	This study
pUC18T-mini-Tn7T-Gm	Mini-Tn7 vector for chromosomal insertion at the *att*Tn7 site, Gm^R^	[69]
pGW-*dtpC*-GM	*dtpC* on pUC18T-mini-Tn7T-Gm	This study
pJF-Tn7T-*dtpC*-STOP-*katE*	*dtpC* 40C > T and *katE* on pUC18T-mini-Tn7T-Gm	This study
pGW-*dtpCkatE*-GM	*dtpC-katE* on pUC18T-mini-Tn7T-Gm	This study
pUC18T-Tn7T-*dtpC*-E143A	*dtpC* 428–429AG > CA on pUC18T-mini-Tn7T-Gm	This study
pJF-Tn7T-*dtpC*-H153A	*dtpC* 457–458CA > GC on pUC18T-mini-Tn7T-Gm	This study
pUC18T-Tn7T-*dtpC*-H232A	*dtpC* 694–695CA > GC on pUC18T-mini-Tn7T-Gm	This study
pSP-Trc	*E. coli-A. baumannii* expression plasmid, *trc* promoter, *lacI*, Gm^R^	This study
pSP-Trc-katE	pSP-Trc expression plasmid carrying *katE*, Gm^R^	This study
		

We also noticed some effects from the very low humidity conditions used in these experiments on the ATCC 17961 Δ*dtpC* mutant strain at normal oxygen levels that we did not observe at 42% RH. The ATCC 17961 Δ*dtpC* mutant strain appeared to be more sensitive to drying at < 5% RH (Fig 3A, +), which was different from the results at 42% RH, where we did not observe any significant decrease in the survival of the Δ*dtpC* mutant strain (Fig 1E). We also quantified the survival of the Δ*dtpC* mutant after drying at < 5% RH, with normal oxygen levels, by counting CFU. This confirmed that the Δ*dtpC* mutant had reduced survival compared to the wild-type ATCC 17961 (S3 Fig). However, the drying survival of the Δ*dtpC* mutant in strain AB5075 did not appear to differ from the wild-type at < 5% RH (Fig 3B) in multiple trials. These results suggested that the importance of *dtpC* for desiccation tolerance may vary depending on the strain or environmental conditions. Notably, the very low humidity conditions tested here would be uncommon in most natural situations.

Since the ability to survive drying improved for all the strains tested, including the wild-type strains ATCC 17961 and AB5075, when oxygen was absent, we wondered if limiting oxygen would also improve the desiccation tolerance of less tolerant strains. To test this, we analyzed the survival of four moderately desiccation-tolerant *A. baumannii* strains, ATCC 17978, A118, ATCC 19606, and AB09−002 [13] after incubating the dried cells in both the presence and absence of oxygen. We observed that none of the strains could survive 14 days of extremely dry conditions with normal oxygen levels, but all four could survive in the absence of oxygen (Fig 3C). These results implied that oxygen-mediated damage generally affects *A. baumannii* cells during desiccation, and that individual strains may vary in their ability to deal with these effects.

The goal of incubating dried cells in the anaerobic chamber was to limit the formation of ROS during desiccation, but it is possible that limiting oxygen affected the dried cells in other ways, such as limiting metabolism. Another way to test the importance of *dtpC* and *katE* in protecting cells from desiccation-induced oxidative stress would be to enhance ROS production. Growing cells under oxygen-saturating conditions can increase ROS levels in cells [49], so we tested to see whether incubating dried cells in the presence of increased oxygen levels would affect drying survival. These experiments were performed in a similar way to the previous tests where oxygen was removed, but instead, paired samples of dried cells were stored with either normal atmospheric oxygen levels, or were put in a container that was flushed with 100% oxygen prior to being sealed. In multiple trials we observed that the survival of the wild-type strain ATCC 17961 and the Δ*dtpC* and Δ*katE* mutant strains all appeared to decrease slightly when exposed to elevated oxygen during desiccation (Fig 3D), but these differences appeared to be minor, and it was not clear if they were significant. In contrast, there was clearly a larger difference in the survival of the Δ*dtpC*Δ*katE* mutant strain, which appeared to have an approximately 100-fold decrease in survival under the increased oxygen conditions (Fig 3D). These results showed that dried *A. baumannii* cells were more sensitive to the detrimental effects of oxygen when both *dtpC* and *katE* were absent.

**Fig 4:**
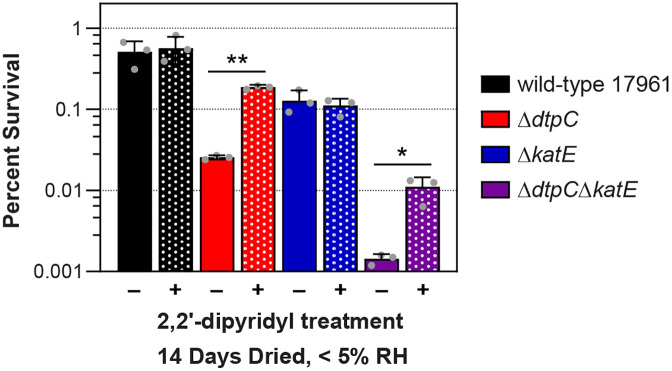
Treatment with the iron chelator 2,2’-dipyridyl improves the survival of Δ*dtpC* mutant strains during desiccation. Cells from the wild-type strain ATCC 17961 and the Δ*dtpC*, Δ*katE*, and Δ*dtpC*Δ*katE* deletion mutants were washed in water and treated either with solvent (solid bars) or 100 µM 2,2’-dipyridyl (dotted bars) prior to drying. The dried cells were incubated at 25°C and < 5% RH for 14 days before resuspension, and survival was assessed by CFU counts. The data presented represent the mean ± SD from three independent experiments. The survival of solvent-treated versus dipyridyl-treated cells was compared for each strain by the Welch’s t-test. ^*^p < 0.05, ^**^p < 0.01.

There have not been any studies to specifically investigate the chemical or biochemical processes that are involved in ROS formation in dried cells, but in aqueous environments free iron is an important contributor. Iron can react with hydrogen peroxide and superoxide via the Fenton and Haber-Weiss reactions, leading to the formation of damaging oxygen radicals [50]. Cell-permeable chelators, such as 2,2’-dipyridyl, can sequester free iron to limit these processes, and treatment of cells with 2,2’-dipyridyl can protect cells from damage by hydrogen peroxide [50]. Therefore, we tested to see whether treatment with 2,2’-dipyridyl would provide any protection to the Δ*dtpC* and Δ*katE* mutant strains during drying. For these experiments cells were grown and washed according to our normal desiccation tolerance assay protocol, and then 100 µM 2,2’-dipyridyl was added to samples prior to drying. The dried cells were incubated at < 5% RH, a condition where the ATCC 17961 Δ*dtpC* mutant had reduced survival (Fig S3), so that we could assess the effects of 2,2’-dipyridyl treatment on the survival phenotypes of both the Δ*dtpC* and Δ*dtpC*Δ*katE* mutant strains. 2,2’-dipyridyl treatment did not affect the survival of the wild-type strain ATCC 17961 or the Δ*katE* mutant, but it significantly improved the survival of the Δ*dtpC* and Δ*dtpC*Δ*katE* mutant strains (Fig 4). Notably, treating the cells with 2,2’-dipyridyl only during the rehydration phase, instead of treating the cells prior to drying, did not have any protective effect (S4 Fig). Taken together, these results suggest that *dtpC* has some role in preventing damage from iron-dependent processes during the period of desiccation. In combination with the data in Fig 3, these results provide further support for the hypothesis that the main protective function of DtpC and KatE in desiccation tolerance is to combat desiccation-induced oxidative stress.

### DtpC and KatE prevent the accumulation of endogenous hydrogen peroxide during desiccation

To protect themselves from the damaging effects of oxidative stress, cells employ a variety of factors that act in different ways. Some factors, such as catalases, scavenge reactive oxygen species before damage occurs, other factors act to prevent ROS formation, and there are also factors that can repair oxidative damage to cellular constituents. Our data thus far do not provide any insight into how DtpC might act to protect cells from desiccation-induced oxidative stress. However, it is logical to assume that as a catalase, KatE provides protection from desiccation by scavenging hydrogen peroxide. This implies that hydrogen peroxide is produced in dried cells. To confirm that this is the case, and as a starting point to learn more about DtpC’s protective activity, we decided to try to assess the levels of hydrogen peroxide in dried cells. In aqueous conditions, endogenously produced hydrogen peroxide can diffuse out of cells into the surrounding medium where it can be measured, although cellular scavenging enzymes typically degrade the hydrogen peroxide before it can exit the cell [51]. For dried cells, we theorized that apart from some evaporative loss, any hydrogen peroxide produced would remain cell-associated. Upon rehydration, we expected that any accumulated hydrogen peroxide would diffuse into the suspending medium, where it could then be measured. For these experiments cell samples were prepared and dried as in our previous experiments, and dried cells were incubated at 25°C and a normal relative humidity of 42%. To measure hydrogen peroxide, cells were allowed to rehydrate in normal saline for three minutes, and then the cells were removed by centrifugation and the amount of hydrogen peroxide present in the saline was assessed using Amplex UltraRed. When hydrogen peroxide is present Amplex UltraRed is oxidized to a fluorescent form via the catalytic activity of horseradish peroxidase. This assay can detect low levels of hydrogen peroxide in aqueous solutions [52], and through measurements of standard solutions we found that we could quantify hydrogen peroxide concentrations down to 50 nM (S5 Fig).

**Fig 5:**
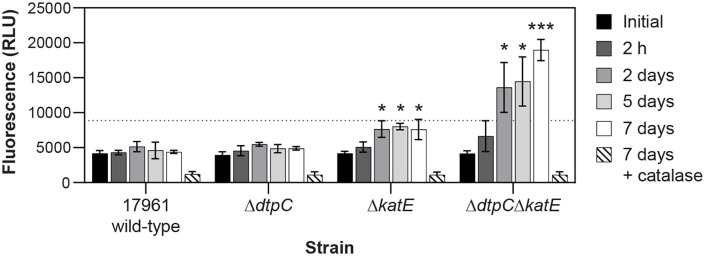
Either *dtpC* or *katE* can prevent the accumulation of hydrogen peroxide in dried cells. Cells from the wild-type strain ATCC 17961 and the Δ*dtpC*, Δ*katE*, and Δ*dtpC*Δ*katE* deletion mutants were dried and incubated at 25°C and 42% RH. At the indicated time points cells were rehydrated in saline for 3 min, then cells were removed by centrifugation and the amount of hydrogen peroxide in the supernatant was assessed based on the conversion of Amplex UltraRed to a fluorescent compound. For a duplicate set of samples at day 7, 16 U of bovine catalase was added to the supernatant prior to the Amplex UltraRed assay as a control (diagonal lines). The dotted line indicates the fluorescence produced by 50 nM hydrogen peroxide, which is the limit of quantification for hydrogen peroxide in this assay. The data presented represent the mean ± SD from four independent experiments. The fluorescence produced by samples from each strain, excluding catalase-treated controls, were compared to wild-type at the same time point by the repeated measures two-factor ANOVA with Dunnett’s multiple comparisons post-test. ^*^p < 0.05, ^***^p < 0.001.

When we analyzed samples from the wild-type strain ATCC 17961 and the Δ*dtpC* mutant strain, we observed that the fluorescence produced remained low over the time course, with the fluorescence measured from dried cells remaining similar to the initial measurements taken from cell suspensions prior to drying (Fig 5). This was not surprising since KatE is present in these cells, which should keep hydrogen peroxide levels low. Samples from the Δ*katE* mutant strain produced slightly increased fluorescence after two days of drying, however these measurements remained too low to be able to quantify hydrogen peroxide (Fig 5). To ensure that these fluorescence readings represented hydrogen peroxide in the samples, and were not due to the reaction of other compounds released from the cells with Amplex UltraRed, we also tested control samples that were treated with catalase after the cells were removed, and immediately prior to the Amplex UltraRed assay. Catalase-treated samples only produced background levels of fluorescence (Fig 5 and S5 Fig), confirming that the fluorescence produced was from hydrogen peroxide. Most interestingly, when we analyzed samples from the Δ*dtpC*Δ*katE* mutant strain, we found that the fluorescence increased as the cells were dried for longer periods of time, with the fluorescence after seven days being approximately four times higher than the wild-type (Fig 5). By comparison with standards, these readings corresponded to about 0.25 µM hydrogen peroxide in samples from the Δ*dtpC*Δ*katE* mutant strain after seven days of drying. However, we measured the hydrogen peroxide concentration present after the cells were resuspended in a volume of 100 µl, which was much larger than the volume occupied by the cells in a dried state. We do not know the exact volume that the dried cells occupied prior to rehydration, but we estimate that it was less than 5 mm^3^. Taking the increase in volume that occurred during rehydration into account, the concentration of hydrogen peroxide in the dried cells would be at least 20 times higher, and we estimate that after seven days the dried Δ*dtpC*Δ*katE* mutant cells contained approximately 5 µM hydrogen peroxide, whereas in the other strains hydrogen peroxide levels were < 1 µM. We note that to confirm this estimate a different experimental approach would be needed to more accurately measure the volume or mass of dried cells. Nevertheless, our data clearly indicate that either DtpC or KatE are sufficient to limit hydrogen peroxide levels in dried cells.

It is also possible that hydrogen peroxide in the samples was generated during the process of rehydration. We believe that this is unlikely for a couple of reasons. First, for this to be true there would have to be a very robust process to generate hydrogen peroxide specifically in the Δ*dtpC*Δ*katE* mutant cells during rehydration in order to cause the large loss in viability that we observed in our desiccation tolerance assays (Fig 1). Second, we found that incubating dried Δ*dtpC*Δ*katE* mutant cells without oxygen was protective (Fig 3), even though the cells were rehydrated and plated in the presence of oxygen for those experiments. This implied that oxygen caused damage mainly during the period of desiccation, instead of during rehydration. Therefore, we believe that the most likely conclusion is that endogenously produced hydrogen peroxide was able to build up over time in the Δ*dtpC*Δ*katE* mutant strain while the cells were in a desiccated state, and that the presence of either *dtpC* or *katE* was able to prevent this accumulation.

**Fig 6:**
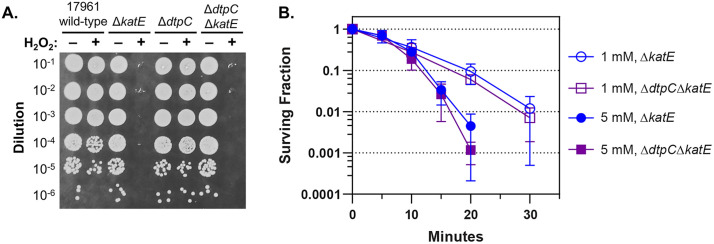
DtpC does not protect cells from acute exposure to hydrogen peroxide. **(A)** Samples of cells from the wild-type strain ATCC 17961 and the Δ*dtpC*, Δ*katE*, and Δ*dtpC*Δ*katE* deletion mutants were dried briefly, and then exposed to 1.5% hydrogen peroxide for 90 sec. After exposure, the hydrogen peroxide was neutralized with 3% sodium thiosulfate, and cells were diluted and plated onto LB media to assess survival. The data presented are representative of three independent experiments. **(B)** Cells from overnight cultures of either the Δ*katE*, deletion mutant (circles) or the Δ*dtpC*Δ*katE* deletion mutant (squares) were washed, suspended in PBS at an equal density, and exposed to either 1 mM (open symbols) or 5 mM (filled symbols) hydrogen peroxide. After exposure, hydrogen peroxide was neutralized with 1% sodium thiosulfate, and survival over time was assessed by CFU counts. The data presented represent the mean ± SD from three independent experiments.

These results suggested that DtpC and KatE have similar roles with regard to desiccation tolerance. We previously found that KatE was able to protect stationary-phase *A. baumannii* cells from challenge with exogenous hydrogen peroxide [13], and we wondered if DtpC could provide similar protection. First, we air-dried bacterial samples on a plastic surface, and then challenged the dried cells with 1.5% (490 mM) hydrogen peroxide. This challenge was similar to the process of disinfecting a non-porous surface with hydrogen peroxide [53]. The wild-type strain ATCC 17961 was able to withstand this treatment, and the deletion of *dtpC* did not appear to affect susceptibility (Fig 6A). In contrast, strains that had *katE* deleted were highly susceptible to this treatment. These experiments challenged cells with a relatively high concentration of hydrogen peroxide (490 mM) for a brief period, but we also tested lower hydrogen peroxide concentrations in aqueous solution. For these tests, we only compared the survival of the Δ*katE* mutant strain, which has *dtpC*, to the Δ*dtpC*Δ*katE* mutant strain, because the robust catalase activity of KatE would likely mask any phenotype in the Δ*dtpC* mutant strain. When we challenged cells with either 1 mM or 5 mM hydrogen peroxide, we did not observe any difference in survival between the Δ*katE* and the Δ*dtpC*Δ*katE* mutant strains (Fig 6B). These results showed that KatE can protect *A. baumannii* cells from high levels of exogenous hydrogen peroxide, but DtpC does not have this same protective capacity, and they suggest that DtpC’s role may be to support long-term persistence in the environment rather than to protect cells from acute oxidative assaults, such as the ROS produced by the respiratory burst of immune cells during infections.

### DtpC and KatE prevent reactive oxygen-associated damage to dried cells

**Fig 7:**
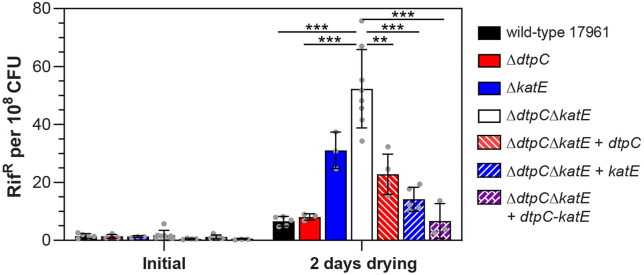
The Δ*dtpC*Δ*katE* mutant strain has an increased frequency of mutations after desiccation. The frequency of rifampicin resistant colonies was determined either prior to desiccation, or after two days of desiccation, for the wild-type strain ATCC 17961; the Δ*dtpC*, Δ*katE*, and Δ*dtpC*Δ*katE* deletion mutants; and the Δ*dtpC*Δ*katE* deletion mutant complemented by insertion of either *dtpC, katE,* or *dtpC-katE* onto the chromosome. The bars represent the mean ± SD from at least three independent experiments, and individual replicates are indicated by gray dots. The rifampicin resistance frequency was compared at each time point by the Welch’s ANOVA with Dunnett’s multiple comparisons post-test. ^**^p < 0.01, ^***^p < 0.001.

If DtpC and KatE preserve the viability of dried cells by limiting the levels of reactive oxygen species, then we would expect that reactive oxygen-associated damage to dried cells would also be limited as long as either *dtpC* or *katE* are present. DNA damage is often observed after desiccation, and is thought to be a result of the oxidative stress that occurs during drying [21]. One consequence of DNA damage is that it can lead to mutations, and mutation frequencies can be used to assess the extent of DNA damage. Notably, the frequency of spontaneous mutations conferring resistance to rifampicin was found to increase after desiccation for *A. baumannii* strains ATCC 17978 and ATCC 19606 [33]. This desiccation-induced increase in rifampicin resistance was dependent on the SOS DNA damage response [33], providing further evidence that drying-induced mutations in *A. baumannii* are generated in response to DNA damage. With this in mind, we compared the frequency of rifampicin resistance for the wild-type strain ATCC 17961; the Δ*dtpC*, Δ*katE*, and Δ*dtpC*Δ*katE* mutant strains; and the Δ*dtpC*Δ*katE* mutant strain complemented with *dtpC, katE,* or *dtpC* and *katE*. Prior to drying, we observed that the rifampicin resistance frequency for all of the strains was similar (Fig 7). After two days of desiccation, the frequency of resistance increased by about 4.5-fold for the wild-type strain ATCC 17961, and we observed a similar increase for the Δ*dtpC* mutant strain (Fig 7). In contrast, there was a larger increase in the frequency of resistance for the Δ*katE* mutant strain after drying, but the largest average increase was observed for the Δ*dtpC*Δ*katE* mutant strain, which had an approximately 30-fold increase in the frequency of resistance to rifampicin (Fig 7). We noticed that these results followed a similar trend to the measurements of hydrogen peroxide released by these strains after two days of drying, with the highest levels of hydrogen peroxide being released from the Δ*dtpC*Δ*katE* mutant strain (Fig 5). Most interestingly, there was less of an increase in rifampicin resistance when the Δ*dtpC*Δ*katE* mutant strain was complemented with either *dtpC* or *katE*, and complementation with both *dtpC* and *katE* lowered the resistance frequency to a wild-type level (Fig 7). These results showed that either *dtpC* or *katE* can limit desiccation-induced mutagenesis, which occurs in response to DNA damage in *A. baumannii* [33], and they support the idea that both DtpC and KatE act to protect cells from the damaging effects of oxidative stress during desiccation.

## Discussion

The natural process of desiccation involves different steps that present multiple challenges to cells. During the initial phase of drying, water evaporates until only a thin layer of water covers the cells, and solutes in the suspending medium become concentrated, potentially creating osmotic stress. As evaporation continues, water begins to be lost from cells, and this is thought to progress until the water content within the cells reaches equilibrium with the water vapor in the surrounding air [21,54]. This results in a loss of cellular volume, and this is evident for desiccated *Acinetobacter* cells examined by electron microscopy, which appear flattened and more electron-dense [27,55]. Cells can respond to these changes during the initial stages of drying, but eventually the water content within the cell decreases to a point where many enzymes cannot function, and metabolism is interrupted [21,54]. Multiple factors are needed to contend with the increased osmolarity, molecular destabilization due to water loss, and other physical and chemical stresses that occur during the different stages of drying. Interestingly, highly desiccation-tolerant organisms often seem to possess multiple protective factors that appear to be functionally similar or redundant [56]. This seems to be the case for *A. baumannii* as well, and our results showed that DtpC and KatE are co-transcribed factors in *A. baumannii* that can each individually protect cells from drying by limiting desiccation-induced oxidative stress.

In addition to *dtpC* and *katE*, *A. baumannii* encodes an array of other factors to defend against oxidative stress. Some of these factors are controlled in a reactive oxygen-inducible manner by the transcriptional regulator OxyR [57]. These factors include a second KatE homolog that is present in strain ATCC 17978 (encoded by gene *A1S_3382*) and some other *A. baumannii* strains, but which is not present in strains ATCC 17961 or AB5075. It is unclear if these reactive oxygen-inducible factors are involved in desiccation tolerance. Instead, a proteomics analysis of dried *A. baumannii* cells showed increased levels of a different set of oxidative stress defenses, including the KatE catalase discussed in this study, an AhpC-like peroxiredoxin, and a Cu, Zn superoxide dismutase (SodC) [27]. Additionally, *katE* transcription was found to be induced by desiccation in *A. baumannii* strain ATCC 17978, and both strains ATCC 17978 and AB5075 were more resistant to killing by hydrogen peroxide after drying [35], implying that the stress of desiccation induces oxidative stress defenses in *A. baumannii.* The expression of *katE, ahpC,* and *sodC* are controlled by the two-component response regulator BfmR, and these factors are all likely induced as part of the general stress response in *A. baumannii* [35,36]*.* DtpC and KatE expression also appear to be positively regulated by the RNA binding protein CsrA [34], suggesting that CsrA may be involved in controlling protective responses in *A. baumannii*. However, only a few studies have examined oxidative stress responses in *A. baumannii*, and further work is needed to understand which oxidative stress defenses are important for environmental persistence, and which are important for survival within a host during infection.

Our findings build on previous evidence that desiccated bacterial cells experience oxidative stress. Some of this evidence is indirect, such as the fact that signs of DNA damage, which is often associated with oxidative stress, are frequently observed during desiccation [21]. Also, oxidative stress defenses were found to be upregulated during desiccation stress in some bacterial species [25–29]. Based on the fact that KatE had a known function in protecting *A. baumannii* from oxidative stress, we decided to test the damaging effects of oxygen during desiccation more directly by incubating dried cells either in the absence of oxygen, or in the presence of elevated oxygen levels. We observed that oxygen was detrimental to the survival of dried *A. baumannii* cells for all of the strains we tested (Fig 3). Similarly, incubation of dried *Bradyrhizobium japonicum* cells under low-oxygen conditions improved survival during desiccation [58]. We also found that the deletion of *dtpC* and *katE* in combination made *A. baumannii* more sensitive to the effects of oxygen during drying (Fig 3), suggesting that DtpC and KatE protect *A. baumannii* cells from oxidative stress during desiccation. Comparably, drying survival of *S. aureus* was reduced when the *katA* and *ahpC* genes, which both encode factors that scavenge hydrogen peroxide, were deleted in combination [59]. Together, these experiments showed that oxidative stress defenses can support desiccation tolerance in multiple bacterial species.

For further confirmation that oxidative stress occurs during desiccation, we measured the amount of hydrogen peroxide released from dried *A. baumannii* cells immediately after rehydration. Data from a recent study suggested that there were elevated levels of reactive oxygen species in *A. baumannii* cells after desiccation using an assay based on the fluorescence of the probe 2,7-dichlorofluorescein diacetate (DCFDA) [60]. However, changes in cell size and membrane permeability were also observed for the dried cells in that study, and these factors can skew the results of the DCFDA assay [61,62]. We utilized the Amplex Ultrared assay, which is a sensitive and well-established method to quantify hydrogen peroxide released from cells [52]. Our results showed that dried *A. baumannii* cells carrying either *dtpC* or *katE* contained low levels of hydrogen peroxide, but increasing amounts of hydrogen peroxide were released from cells of a Δ*dtpC*Δ*katE* mutant strain as the cells were dried for longer periods of time (Fig 5). Based on our measurements, we estimated that the hydrogen peroxide concentration in the dried cells of the Δ*dtpC*Δ*katE* mutant strain reached the low micromolar range after seven days. This level of hydrogen peroxide is typically too low to cause significant damage to cells due to the activity of the cellular oxidative stress defenses [63], but it can be enough to cause damage if these defenses are compromised. In *E. coli,* DNA repair mutants that are exposed to low micromolar concentrations of hydrogen peroxide for an extended time have decreased viability, and show signs of DNA damage [63]. Considering that the activity of many enzymes, including DNA repair functions, are likely compromised in desiccated cells, it is plausible that the presence of low levels of hydrogen peroxide and other reactive oxygen species can lead to significant cellular damage in dried cells over time. In support of this idea, we found that there was an increased mutation rate in the Δ*dtpC*Δ*katE* mutant strain after drying compared to the wild-type (Fig 7), suggesting that more DNA damage occurs when DtpC and KatE are not present to protect dried cells. Our results agree with previous findings that mutation frequency in *A. baumannii* is elevated after desiccation [33], and they suggest that oxidative stress is involved in desiccation-induced mutagenesis. Overall, our data support the idea that desiccation is a natural condition where bacteria are exposed to oxidative stress, and that oxidative stress defenses are key factors needed for bacteria to survive in a dried state.

Additionally, we found that sequestering free iron with the chelator 2,2’-dipyridyl improved the drying survival of Δ*dtpC* mutant strains (Fig 4). The main contribution of iron to oxidative stress in biological systems is through Fenton chemistry, whereby hydrogen peroxide, which is not particularly reactive on its own, is converted to damaging hydroxyl radicals through its reactions with free iron [50]. Iron can also contribute to oxidative stress through the formation of ferryl radicals that can directly damage DNA [50]. Our results imply that in the absence of *dtpC*, dried cells are damaged through an iron-dependent process. However, since we also observed that DtpC limits hydrogen peroxide production in dried cells (Fig 5), it is not clear whether DtpC is directly involved limiting iron-mediated damage (which could be through its ability to bind and sequester free iron), or if it acts to lower the levels of the oxygen species present that are reacting with the available iron.

While it is not surprising to find that iron is involved in desiccation-induced oxidative stress, the chemical or biochemical processes that generate reactive oxygen species in dried cells, or what types of reactive oxygen species other than hydrogen peroxide are being generated, are currently unknown. It is interesting to note that the chemistry of reactive oxygen species at the air-water interface can differ from reactions that occur in the bulk aqueous phase [64]. Therefore, the processes that drive the formation of reactive oxygen species in dried cells may be different from fully hydrated cells in an aqueous environment. With this in mind, it is not possible to propose a specific mechanism for DtpC’s protective activity based on our current data. However, we can consider some possibilities based on the activities of other factors that protect cells from oxidative stress. As we already mentioned, one possibility is that DtpC prevents iron-mediated oxidative damage by sequestering free iron. DtpC is predicted to bind two iron atoms, and mutating amino acid residues involved in metal binding disrupted its protective activity (Fig 2). These mutations also would have disrupted DtpC’s potential catalytic activity, and so it is unclear whether its protective function involves iron sequestration, or if protection is due to its enzymatic activity. Additionally, it is possible that DtpC may bind other metals in its active site instead of iron. The HDO enzyme CADD binds one iron and one manganese atom in its active site instead of two iron atoms [43]. A second possibility is that DtpC acts to scavenge reactive oxygen species. Our data showed that DtpC does not appear to protect cells from acute hydrogen peroxide stress (Fig 6), but hydrogen peroxide in dried cells is likely produced at a slow rate, and then builds up over time. Therefore, DtpC could potentially be able to scavenge hydrogen peroxide efficiently enough to protect dried cells, yet still be incapable of protecting cells from an acute challenge. Alternatively, DtpC could act to scavenge a different reactive oxygen species such as superoxide, which can be reduced to form hydrogen peroxide. A third intriguing possibility is that DtpC could be responsible for the biosynthesis of a protective small molecule. Small molecules such as glutathione can play important roles in protecting cells from oxidative stress. Based on structural predictions and mutational analysis (Fig 2), DtpC is a HDO-family enzyme, and these enzymes often appear to be involved in the biosynthesis of small molecules, sometimes through novel catalytic mechanisms [65]. Importantly, no other HDO family members have been linked to oxidative stress defense or desiccation tolerance, making DtpC a new type of protective factor. Learning more about DtpC’s catalytic activity will help to clarify its specific protective mechanism, and may provide new insight into desiccation-induced oxidative stress. The ability to persist on dry surfaces is a trait that facilitates the spread of *A. baumannii* in hospitals, and understanding the mechanisms that contribute to desiccation tolerance may lead to new strategies to prevent infections by this challenging opportunistic pathogen.

## Materials and methods

### Bacterial strains and growth conditions

The bacterial strains used in this study are listed in Table 1. Frozen stocks of *Escherichia coli* and *A. baumannii* strains containing either 15% glycerol or 10% skim milk were stored at −80˚C, and bacteria were freshly plated from frozen stocks to begin each experiment. Bacteria were cultured in lysogeny broth (LB; Lennox formulation), and were grown at 37˚C. Broth cultures were incubated with shaking at 240–260 rpm. When necessary for molecular cloning or mutation procedures, or to maintain plasmids, the growth medium was supplemented with antibiotics at the following concentrations: For *E. coli*, 100 µg/ml ampicillin, 10 µg/ml gentamicin, 10 µg/ml tetracycline, and 50 µg/ml kanamycin; for *A. baumannii*, 150 µg/ml carbenicillin, 20 µg/ml gentamicin, and 5–10 µg/ml tetracycline.

### Reverse transcription-polymerase chain reaction (RT-PCR) analysis

To isolate RNA for RT-PCR analyses, cells from an overnight culture of *A. baumannii* strain ATCC 17961 were used to inoculate LB medium to an optical density at 600 nm (OD_600_) of 0.05, and this culture was incubated at 37°C for 6 h. Then the culture was treated with RNAprotect Bacteria Reagent (Qiagen), and cells were harvested by centrifugation. Cells were incubated in lysis buffer (30 mM Tris pH 8, 1 mM EDTA, 10 mg/ml lysozyme, 2 mg/ml proteinase K) for 5 minutes at room temperature, and then RNA was isolated using TRIzol Reagent (Life Technologies). RNA samples were treated with TURBO DNase (Life Technologies) prior to RT-PCR analysis. RT-PCR was performed using the SuperScript III One-Step RT-PCR System (Life Technologies) with 500 ng of RNA as a template using the oligonucleotide primers listed in Table S1. Control reactions were performed that did not include reverse transcriptase to confirm that the RNA samples did not have any DNA contamination. Equal volumes of each completed reaction were analyzed by electrophoresis on an agarose gel.

### Construction of plasmids

For the construction of plasmids to delete both *dtpC* and *katE*, approximately 1 kb DNA fragments upstream from *dtpC* and downstream from *katE* were amplified by PCR using chromosomal DNA from either strain ATCC 17961 or strain AB5075 as a template. The oligonucleotide primers for these reactions were designed to add either HindIII and EcoRI (upstream fragment) or EcoRI and BamHI (downstream fragment) (S1 Table). These DNA fragments were digested with the appropriate enzymes, purified after agarose gel electrophoresis, and ligated with either vector plasmid pEX18Ap, or pEX18Tc, that had been digested with HindIII and BamHI to generate plasmids pΔ*dtpCkatE*-suc and pTcΔ*dtpCkatE*-suc, respectively. To construct plasmids for the deletion of *dtpC* or *katE* individually, mutant alleles containing in-frame deletions for both genes were generated using a splicing by overlap extension PCR technique [70] with the primers listed in S1 Table. The resulting PCR fragments were designed to remove the DNA coding sequenced for amino acids 8–463 of DtpC (97% of the protein sequence) or 41–680 of KatE (90% of protein sequence), and restriction sites were added to each end of the fragments to facilitate cloning. Each PCR fragment and either pEX18Ap or pEX18Tc were digested with the appropriate enzymes and ligated to produce plasmids pΔ*dtpC*-suc, pTcΔ*dtpC*-suc, and pTcΔ*katE*-suc.

To generate constructs for the insertion of *dtpC* and *katE* onto the bacterial chromosome at the *att*Tn7 site, PCR products were amplified that spanned from 349 bp upstream from the *dtpC* translational start site to either immediately downstream from the *dtpC* stop codon for *dtpC* alone, or immediately downstream from the *katE* stop codon for *dtpC-katE*. Oligonucleotide primers for these amplifications were designed to add restriction sites at the end of each fragment to facilitate cloning. These fragments were digested with appropriate enzymes and ligated with pUC18T-mini-Tn7T-Gm, which had been digested with the same enzymes, to produce plasmids pGW-*dtpC*-GM and pGW-*dtpCkatE*-GM. Plasmid pGW-*dtpCkatE*-GM was used as a template for PCR with oligonucleotide primers dtpC STOP F and dtpC STOP R (S1 Table), and the resulting fragment was circularized by ligation to produce plasmid pJF-Tn7T-*dtpC*-STOP-*katE*. This plasmid has a stop codon introduced into the *dtpC* coding sequence at codon 14, in order to block the translation of *dtpC* while allowing for *katE* expression from its natural promoter. The same approach was used to generate plasmids pJF-Tn7T-*dtpC*-H153A, pUC18T-Tn7T-*dtpC*-E143A, and pUC18T-Tn7T-*dtpC*-H232A, but instead different primer sets were used (S1 Table) with plasmid pGW-*dtpC*-GM as a template.

For cloning an expression plasmid for use in *A. baumannii*, plasmid pTrcHis-A (Invitrogen) was used as a template for PCR with primers TrcHis InvF and TrcHis InvR, in order to remove the His-tag coding sequences from the plasmid. The resulting DNA fragment was circularized by ligation to produce pJF-Trc. Next, an approximately 2.3 kb DNA fragment containing the *trc* promoter and *lacI* was amplified by PCR using pJF-Trc as a template. For the remaining portions of the plasmid, pVRL2 was used as a template for PCR with primers pVRL2 nsi inv and pVRL2 sca inv, and the resulting fragment was circularized by ligation to produce pVRL2Δ*araC*pBAD-ScaI. Plasmid pVRL2Δ*araC*pBAD-ScaI was digested with ScaI and ZraI, and then ligated with the DNA fragment carrying *lacI* and P*trc* to produce the *E. coli-A. baumannii* expression vector pSP-Trc. To generate a plasmid for the expression of *katE*, a DNA fragment with the sequences from 32 bp upstream from the *katE* translational start site to the *katE* stop codon was amplified by PCR. The oligonucleotide primers for this amplification were designed to add NcoI sites to each end of the fragment. The DNA fragment was digested with NcoI and ligated with pSP-Trc, which had also been digested with NcoI, to produce pSP-Trc-katE.

### Generation of mutant strains

Mutant strains were generated by allelic exchange using the procedures described previously [13]. The mini-Tn7 system was used to insert genes into the *att*Tn7 site on the *A. baumannii* chromosome for complementation [71]. Genetic regions carried on pUC18T-mini-Tn7-based plasmids, along with pTNS2, were introduced into *A. baumannii* strains by four-parental mating as described by Kumar *et al.* [71], except that 5 µg/ml chloramphenicol was used to select against the *E. coli* strains in the mating mixture instead of streptomycin. The insertion of DNA sequences at the *att*Tn7 site was confirmed by PCR. For plasmid-based complementation, plasmids were introduced into *A. baumannii* strains by electroporation.

### Desiccation tolerance assays

Desiccation tolerance assays were performed as described previously [13]. Briefly, cells from overnight cultures were washed and then suspended in deionized water at an OD_600_ of 1.0. Next, 5 µl samples of these bacterial suspensions were deposited on a polystyrene surface and left uncovered in a biosafety cabinet at room temperature (21–24˚C) to dry. Once the samples were visibly dry, they were stored at 25˚C in the dark at constant relative humidity (RH). For a RH of 42%, samples were stored in a sealed container over a saturated solution of potassium carbonate. For a RH of < 5% samples were stored in a sealed container with Drierite desiccant. To determine the initial number of colony forming units (CFU) present, 5 µl of the initial bacterial suspension was diluted into 95 µl of normal saline (0.9% sodium chloride), and then a five-fold dilution series was made in saline. To determine the CFU present in dried samples, 20 µl of saline was placed on a dried bacterial sample and allowed to sit for 5 min to rehydrate, followed by pipetting and gently scraping of the sample to remove the cells from the plastic surface. The suspended cells were then diluted into 80 µl saline, and a five-fold dilution series was made in saline. After serial dilutions, 20 µl of dilutions were spotted onto LB agar and incubated at 37°C overnight. Duplicate samples were diluted and plated for each strain at each time point. After incubation, the plates were viewed with a dissecting microscope and the number of CFU in each spot was counted. The original CFU present in each sample was calculated based on the dilution factor. For a qualitative assessment of survival, the samples were diluted in saline and spotted onto LB agar as described previously [13].

To test the effect of 2,2’-dipyridyl treatment on desiccation tolerance, 2.5 µl of either 50% ethanol or 10 mM 2,2’-dipyridyl dissolved in 50% ethanol was added to 250 µl of bacterial suspension, which was then used to prepare dried samples. All other steps were performed in the same way as normal desiccation tolerance assays.

### Measurement of hydrogen peroxide

Bacterial samples for the measurement of hydrogen peroxide from dried cells were prepared as described for desiccation tolerance assays. For initial measurements, 80 µl samples of OD_600_-adjusted bacterial suspensions were centrifuged to pellet cells, and the supernatant was removed. The cell pellet was suspended in 100 µl of saline and incubated at room temperature for 3 min. The samples were then centrifuged to pellet the cells, and the supernatant was used immediately for the measurement assay. For measurements from dried cells, 5 µl of saline was deposited onto each of 16 dried bacterial samples, and the samples were allowed to rehydrate at room temperature for 3 min. The samples were then suspended by pipetting and gently scraping to remove bacteria from the plastic surface. The samples were combined into a single microcentrifuge tube, which was centrifuged to pellet cells, and the supernatant was taken to a clean tube. The volume of the supernatant was brought up to 100 µl with saline, and the sample was used immediately for the measurement assay. Duplicate samples were prepared and assayed for each strain at each time point tested. For catalase-treated control samples, 16 U of bovine catalase (Sigma C9322) was added to the sample prior to the measurement assay.

The Amplex UltraRed assay to measure hydrogen peroxide was based on the procedure of Li and Imlay [52]. Solutions of 0.25 mM Amplex UltraRed (Life Technologies) and 50 µg/ml horseradish peroxidase (Sigma), each dissolved in 50 mM potassium phosphate buffer pH 7.8, were freshly prepared for each experiment. For each assay, 100 µl of test sample was transferred into a well of a black-walled, µClear 96 well plate (Greiner). Next, 50 µl of 0.25 mM Amplex UltraRed was added to the well, followed by 50 µl of 50 µg/ml horseradish peroxidase, and the sample was mixed briefly by pipetting. The plate was then immediately loaded into a Tecan Spark microplate reader, and the fluorescence was measured. The excitation and emission wavelengths used were 530 nm and 590 nm, respectively, and a standard gain setting was used for all measurements.

### Hydrogen peroxide sensitivity assays

Hydrogen peroxide sensitivity assays were performed as described previously [13], with some modifications. To test the sensitivity of dried cells to hydrogen peroxide, dried cell samples were prepared as described for desiccation tolerance assays. Samples were allowed to dry on a plastic surface for 2 h. Then dried samples were treated with 10 µl of either deionized water or 1.5% hydrogen peroxide for 90 sec. After this treatment, 10 µl of 3% sodium thiosulfate (STS) was added to each sample to neutralize the hydrogen peroxide, and the bacterial cells were suspended by pipetting. Bacterial suspensions were diluted serially in 3% STS, 10 µl of each dilution was spotted onto the surface of an LB agar plate, and the plate was incubated at 37°C overnight to assess survival.

To test hydrogen peroxide sensitivity in aqueous solution, cells from overnight cultures were washed with warm phosphate buffered saline (PBS) pH 7.0. Washed cells were used to prepare a cell suspension in PBS at an OD_600_ of 0.1, and samples of the cell suspension were taken to determine the initial CFU/ml. A 1 ml aliquot of the cell suspension was transferred to a test tube and incubated at 37°C for 5 min. Then hydrogen peroxide was added to the suspension to a final concentration of either 1 mM or 5 mM, and suspensions were incubated with rotation at 37°C. At subsequent time points samples were taken from each suspension and immediately diluted into 1% STS. Samples were serially diluted in 1% STS, plated onto LB agar, and colony counts were performed as described for desiccation tolerance assays. Duplicate samples were diluted and plated for each strain at each time point.

### Assessment of rifampicin resistance frequency

To measure the frequency of rifampicin resistance (rif^R^) in *A. baumannii* strains before and after desiccation, dried cell samples were prepared as described for desiccation tolerance assays. To measure the initial rif^R^ frequency for each strain before drying, aliquots of the OD_600_-adjusted bacterial suspensions were plated onto LB agar supplemented with 100 µg/ml rifampicin. A sample of the bacterial suspension was also diluted serially and plated on LB agar to determine the CFU/ml, so that the total number of CFU plated onto media with rifampicin could be calculated. For the analysis of dried bacterial samples after two days at 25°C and 42% RH, dried bacterial spots were suspended in 10 µl of saline each using the procedure described for desiccation tolerance assays. For each strain, bacteria from 13 dried spots were used to inoculate 2 ml LB broth, except that for the Δ*dtpC*Δ*katE* mutant strain, bacteria from 26 dried spots were used to account for the loss of viability for this strain after two days of drying. Cultures were incubated at 37°C with agitation until the OD_600_ was 1.0 to 1.5. Then aliquots of the cultures were plated onto LB agar supplemented with 100 µg/ml rifampicin, and culture samples were also diluted serially and plated onto LB agar to determine the CFU/ml. For initial samples a minimum of 1 × 10^9^ total CFU, and for samples post-drying, a minimum of 5 × 10^8^ total CFU was plated onto media with rifampicin. The number of colonies on plates were counted after overnight incubation at 37°C.

### Statistical analysis

Statistical analyses were performed using GraphPad Prism 10.4.1.

## Supporting information

S1 Fig Plasmid-based complementation with *katE* restores desiccation tolerance to the Δ*dtpC*Δ*katE* mutant strain. The wild-type strain ATCC 17961 and the Δ*dtpC*Δ*katE* mutant in strain ATCC 17961 were each transformed with either the empty expression vector pSP-Trc, or the *katE* expression plasmid pSP-Trc-katE. The survival of each strain before and after desiccation at 42% RH was then assessed. These data are representative of two independent experiments.
(PDF)

S2 Fig Original versions of images used in Fig 3. (A) Original versions of images used in Fig 3A. The un-labeled lanes show data from a strain that was omitted from the study due to off-target mutations. (B) Original versions of the images used in Fig 3B. This image shows the original orientation prior to editing.
(PDF)

S3 Fig The importance of *dtpC* and *katE* genes for *A. baumannii* survival under extremely dry conditions. The survival of the *A. baumannii* wild-type strain ATCC 17961, and Δ*dtpC*, Δ*katE*, and Δ*dtpC*Δ*katE* deletion mutants in strain ATCC 17961, was assessed by CFU counts before and after desiccation at < 5% RH. The data presented are the mean ± SD from at least three independent experiments (for Δ*dtpC* and Δ*katE, n* = 3; for the wild-type and Δ*dtpC*Δ*katE, n* = 4). The mean CFU for each strain at each timepoint was compared to the wild-type by Welch’s ANOVA with Dunnett’s multiple comparisons post-test. **p < 0.01, ***p < 0.001.
(PDF)

S4 Fig Treatment with 2,2’-dipyridyl during rehydration only does not improve recovery from desiccation. Cells from the wild-type strain ATCC 17961 and the Δ*dtpC*Δ*katE* deletion mutant were washed in water, dried, and incubated at 25°C and < 5% RH for 14 days before rehydration. For rehydration, cells were suspended in 0.9% NaCl supplemented with either with solvent (solid bars) or 100 µM 2,2’-dipyridyl (dotted bars) prior to dilution and plating to assess CFU counts. The data presented represent the mean ± SD from three independent experiments. The survival of solvent-treated versus dipyridyl-treated cells was compared for each strain by Welch’s t-test. n.s. = not significant.
(PDF)

S5 Fig Analysis of catalase-treated control samples and standards for Amplex UltraRed assays. (A) Cells from the wild-type strain ATCC 17961 and the Δ*dtpC*, Δ*katE*, and Δ*dtpC*Δ*katE* deletion mutants were dried and incubated at 25°C and 42% RH. At the indicated time points cells were rehydrated in saline for 3 min, then cells were removed by centrifugation and the amount of hydrogen peroxide in the supernatant was assessed by the Amplex UltraRed assay. For each timepoint, 16 U of bovine catalase was added to a duplicate set of samples prior to the Amplex UltraRed assay as a control (diagonal lines). The dotted line indicates the fluorescence produced by 50 nM hydrogen peroxide, which is the limit of quantification for hydrogen peroxide in this assay. The data presented represent the mean ± SD from at least three independent experiments. (B) Solutions containing known amounts of hydrogen peroxide in saline were analyzed by the Amplex UltraRed assay. The data presented represent the mean ± SD from twenty independent measurements. A line of best fit was generated by simple linear regression. R^2^ = 0.9566, p < 0.0001.
(PDF)

S1 Table Oligonucleotide primers used in this study. (PDF)
