# Supplementary material for: Acinetobacter baumannii utilizes a novel protective factor to combat desiccation-induced oxidative stress
Source: PLoS One. 2026 Jun 3;21(6):e0350814. doi: 10.1371/journal.pone.0350814 (PMC13232832; doi:10.1371/journal.pone.0350814)
Supplement: S1 Fig — The wild-type strain ATCC 17961 and the ΔdtpCΔkatE mutant in strain ATCC 17961 were each transformed with either the empty expression vector pSP-Trc, or the katE expression plasmid pSP-Trc-katE. The survival of each strain before and after desiccation at 42% RH was then assessed. These data are representative of two independent experiments. (PDF) [file pone.0350814.s001.pdf]

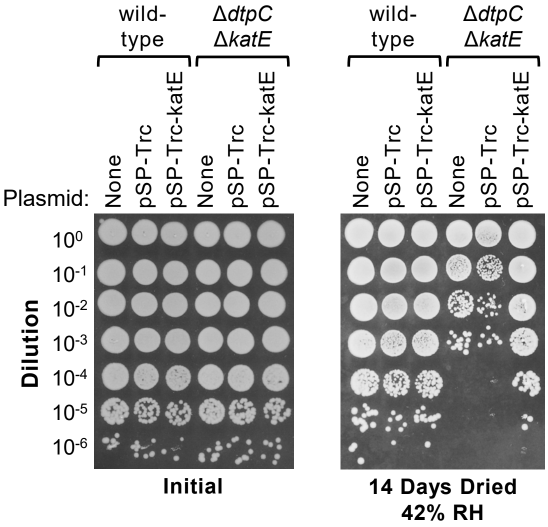

S1 Fig. Plasmid-based complementation with *katE* restores desiccation tolerance to the Δ*dtpC*Δ*katE* mutant strain. The wild-type strain ATCC 17961 and the Δ*dtpC*Δ*katE* mutant in strain ATCC 17961 were each transformed with either the empty expression vector pSP-Trc, or the *katE* expression plasmid pSP-Trc-katE. The survival of each strain before and after desiccation at 42% RH was then assessed. These data are representative of two independent experiments.
