# Supplementary material for: Acinetobacter baumannii utilizes a novel protective factor to combat desiccation-induced oxidative stress
Source: PLoS One. 2026 Jun 3;21(6):e0350814. doi: 10.1371/journal.pone.0350814 (PMC13232832; doi:10.1371/journal.pone.0350814)
Supplement: S2 Fig — (A) Original versions of images used in Fig 3A. The un-labeled lanes show data from a strain that was omitted from the study due to off-target mutations. (B) Original versions of the images used in Fig 3B. This image shows the original orientation prior to editing. (PDF) [file pone.0350814.s002.pdf]

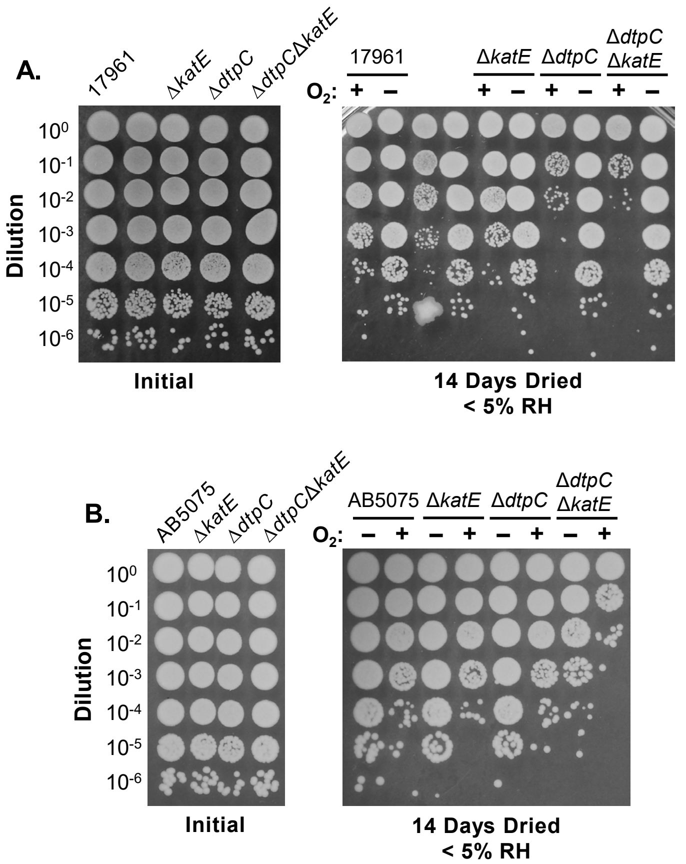

S2 Fig. Original versions of images used in Fig 3. (A) Original versions of images used in Fig 3A. The un-labeled lanes show data from a strain that was omitted from the study due to off-target mutations. (B) Original versions of the images used in Fig 3B. This image shows the original orientation prior to editing.
