# Supplementary material for: Acinetobacter baumannii utilizes a novel protective factor to combat desiccation-induced oxidative stress
Source: PLoS One. 2026 Jun 3;21(6):e0350814. doi: 10.1371/journal.pone.0350814 (PMC13232832; doi:10.1371/journal.pone.0350814)
Supplement: S3 Fig — The survival of the A. baumannii wild-type strain ATCC 17961, and ΔdtpC, ΔkatE, and ΔdtpCΔkatE deletion mutants in strain ATCC 17961, was assessed by CFU counts before and after desiccation at < 5% RH. The data presented are the mean ± SD from at least three independent experiments (for ΔdtpC and ΔkatE, n = 3; for the wild-type and ΔdtpCΔkatE, n = 4). The mean CFU for each strain at each timepoint was compared to the wild-type by Welch’s ANOVA with Dunnett’s multiple comparisons post-test. **p < 0.01, ***p < 0.001. (PDF) [file pone.0350814.s003.pdf]

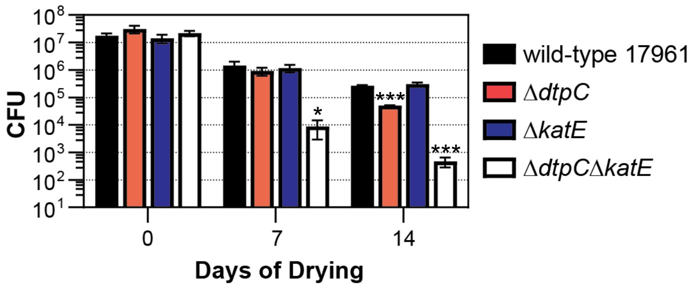

S3 Fig. The importance of *dtpC* and *katE* genes for *A. baumannii* survival under extremely dry conditions. The survival of the *A. baumannii* wild-type strain ATCC 17961, and Δ*dtpC*, Δ*katE*, and Δ*dtpC*Δ*katE* deletion mutants in strain ATCC 17961, was assessed by CFU counts before and after desiccation at < 5% RH. The data presented are the mean ± SD from at least three independent experiments (for Δ*dtpC* and Δ*katE, n* = 3; for the wild-type and Δ*dtpC*Δ*katE, n* = 4). The mean CFU for each strain at each timepoint was compared to the wild-type by Welch's ANOVA with Dunnett's multiple comparisons post-test. **p < 0.01, ***p < 0.001
