# Supplementary material for: Acinetobacter baumannii utilizes a novel protective factor to combat desiccation-induced oxidative stress
Source: PLoS One. 2026 Jun 3;21(6):e0350814. doi: 10.1371/journal.pone.0350814 (PMC13232832; doi:10.1371/journal.pone.0350814)
Supplement: S4 Fig — Cells from the wild-type strain ATCC 17961 and the ΔdtpCΔkatE deletion mutant were washed in water, dried, and incubated at 25°C and < 5% RH for 14 days before rehydration. For rehydration, cells were suspended in 0.9% NaCl supplemented with either with solvent (solid bars) or 100 µM 2,2’-dipyridyl (dotted bars) prior to dilution and plating to assess CFU counts. The data presented represent the mean ± SD from three independent experiments. The survival of solvent-treated versus dipyridyl-treated cells was compared for each strain by Welch’s t-test. n.s. = not significant. (PDF) [file pone.0350814.s004.pdf]

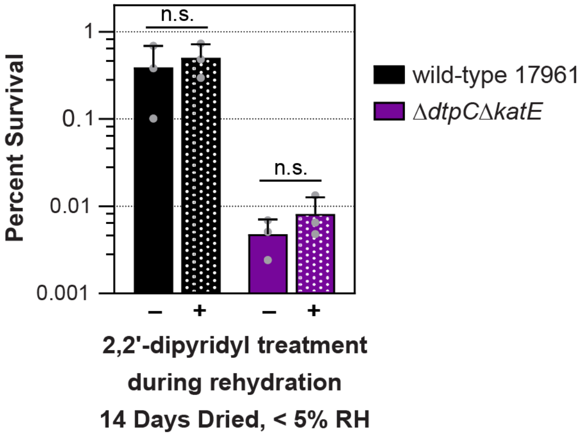

S4 Fig. Treatment with 2,2'-dipyridyl during rehydration only does not improve recovery from desiccation. Cells from the wild-type strain ATCC 17961 and the Δ*dtpC*Δ*katE* deletion mutant were washed in water, dried, and incubated at 25°C and < 5% RH for 14 days before rehydration. For rehydration, cells were suspended in 0.9% NaCl supplemented with either with solvent (solid bars) or 100 µM 2,2'-dipyridyl (dotted bars) prior to dilution and plating to assess CFU counts. The data presented represent the mean ± SD from three independent experiments. The survival of solvent-treated versus dipyridyl-treated cells was compared for each strain by Welch's t-test. n.s. = not significant.
