# Supplementary material for: Acinetobacter baumannii utilizes a novel protective factor to combat desiccation-induced oxidative stress
Source: PLoS One. 2026 Jun 3;21(6):e0350814. doi: 10.1371/journal.pone.0350814 (PMC13232832; doi:10.1371/journal.pone.0350814)
Supplement: S5 Fig — (A) Cells from the wild-type strain ATCC 17961 and the ΔdtpC, ΔkatE, and ΔdtpCΔkatE deletion mutants were dried and incubated at 25°C and 42% RH. At the indicated time points cells were rehydrated in saline for 3 min, then cells were removed by centrifugation and the amount of hydrogen peroxide in the supernatant was assessed by the Amplex UltraRed assay. For each timepoint, 16 U of bovine catalase was added to a duplicate set of samples prior to the Amplex UltraRed assay as a control (diagonal lines). The dotted line indicates the fluorescence produced by 50 nM hydrogen peroxide, which is the limit of quantification for hydrogen peroxide in this assay. The data presented represent the mean ± SD from at least three independent experiments. (B) Solutions containing known amounts of hydrogen peroxide in saline were analyzed by the Amplex UltraRed assay. The data presented represent the mean ± SD from twenty independent measurements. A line of best fit was generated by simple linear regression. R2 = 0.9566, p < 0.0001. (PDF) [file pone.0350814.s005.pdf]

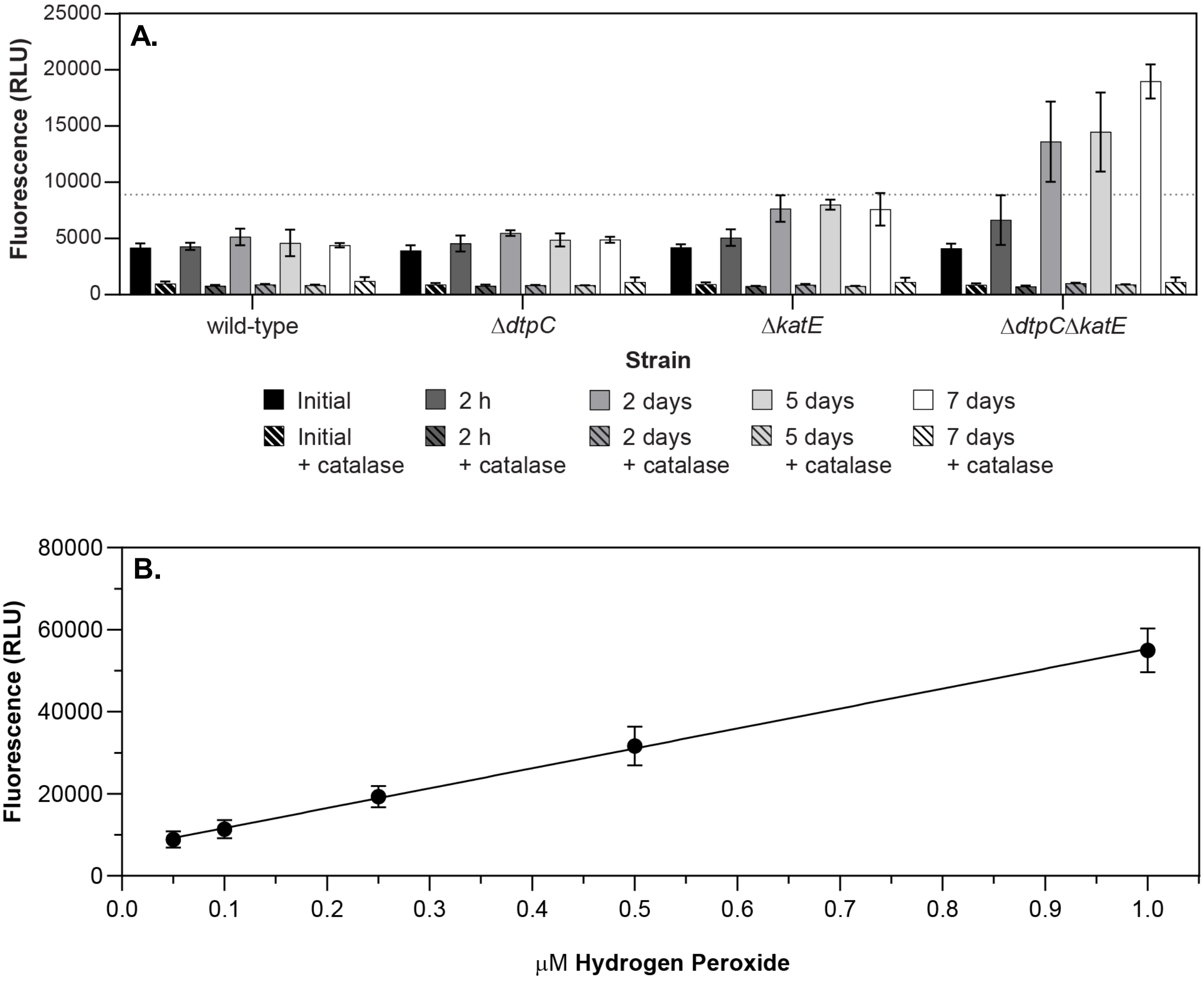

S5 Fig. Analysis of catalase-treated control samples and standards for Amplex UltraRed assays. (A) Cells from the wild-type strain ATCC 17961 and the Δ*dtpC*, Δ*katE*, and Δ*dtpC*Δ*katE* deletion mutants were dried and incubated at 25°C and 42% RH. At the indicated time points cells were rehydrated in saline for 3 min, then cells were removed by centrifugation and the amount of hydrogen peroxide in the supernatant was assessed by the Amplex UltraRed assay. For each timepoint, 16 U of bovine catalase was added to a duplicate set of samples prior to the Amplex UltraRed assay as a control (diagonal lines). The dotted line indicates the fluorescence produced by 50 nM hydrogen peroxide, which is the limit of quantification for hydrogen peroxide in this assay. The data presented represent the mean ± SD from at least three independent experiments. (B) Solutions containing known amounts of hydrogen peroxide in saline were analyzed by the Amplex UltraRed assay. The data presented represent the mean ± SD from twenty independent measurements. A line of best fit was generated by simple linear regression. $R^2 = 0.9566$, $p < 0.0001$.
