# Supplementary material for: Acinetobacter baumannii utilizes a novel protective factor to combat desiccation-induced oxidative stress
Source: PLoS One. 2026 Jun 3;21(6):e0350814. doi: 10.1371/journal.pone.0350814 (PMC13232832; doi:10.1371/journal.pone.0350814)
Supplement: S1 Table — (PDF) [file pone.0350814.s006.pdf]

S1 Table. Oligonucleotide primers used in this study.

Procedure and Primer Name	Sequence (5’→3’)[a, b]
RT-PCR	
katE control (F1)	TGATTTACCTTCAGCATTAATTAA
katE control (R1)	TATACCCAAGAAGGTAACTTCG
katE RT (F2)	TTAAAGCTTCATTAGTTGCATC
dtpC control (F3)	TAATTCAGCAAGCTCATAATT
dtpC control (R3)	TAGACGGTGCATGGCTATATAG
katEdtpC RT (R2)	GGTCATGCCTATAAAGCCATT
dtpC RT (F4)	GTTGATTATCTTGTAATAAGTCT
Generation of mutant alleles	
Del dtpC P1	AAAAAAGGATCCGAAGTCAAAGTTCATTTCATCTT
Del dtpC P2	AACTAGTTTTTAAACGTACCTAATTTTTTCACTACAACAGGCATT
Del dtpC P3	AATGCCTGTTGTAGTGAAAAAATTAGGTACGTTTA AAAACTAGTT
Del dtpC P4	AAAAAAGGATCCAGATTTTAAATGTGTAGGGTA
Del dtpC-katE P1	AAAAAAAAGCTTCCTAACTTGATATGTTATA
Del dtpC-katE P2	AAAAAAGAATTCATAAAAAAAGCCCTGAAAGT
Del dtpC-katE P3	AAAAAAGAATTCTTTCATTGTCCGTTTGAGAG
Del dtpC-katE P4	AAAAAAGGATCCTTTTAATACCTCATATCAAT
5075 del dtpCkatE P2	ACTTTCAGGGCTTTTTTTATTTTCATTCTCCGTTTGTGAG
5075 del dtpCkatE P3	CTCACAAACGGAGAATGAAAATAAAAAAAGCCCTGAAAGT
5075 dtpC INV R	TATTTAGGTACGTTTAAAAACTAG
5075 dtpC INV F	TCGTTTTTTCACTACAACAGGCAT
Cloning for mini-Tn7-based complementation	
Comp dtpC R3	AAAAAACTGCAGTCCTTTGCTAATAATTCGCAACAT
Comp dtpC F3	AAAAAACTGCAGCTAGTTTTTAAACGTACCTAA
katE expr F	AAAAAAGAATTCTTAAGCCGGTACATGTGCGGCT
Expression plasmid cloning	
KatE shuttle R	AAAAAAGGATCCAAGTTTAACTTTCAAAAACA
KatE shuttle F	AAAAAAGTCGACTTAAGCCGGTACATGTGCGG
Site-directed mutagenesis	
dtpC STOP F	TTaTAATAAGTCTAATAATCG
dtpC STOP R	GATAATCAACAAAATTATTATG
dtpC E143A F	GAGATAAGTAATAATTAATTCATG
dtpC E143A R	GAAGcaCTTGGGTTAGGTG
dtpC H153A F	GTTTGCTTTTGGCTCACCTA
dtpC H153A R	GcTGTCTGTATCTATGATGAC
dtpC H232A F	GcAAGATTAAAGTATTTAGAATC
dtpC H232A R	TATCACTATCGACAATATTGAC

[a]Underlined sequence denotes a restriction site utilized for cloning.

[b]Nucleotides shown in lowercase letters are altered compared to the original template sequence.
